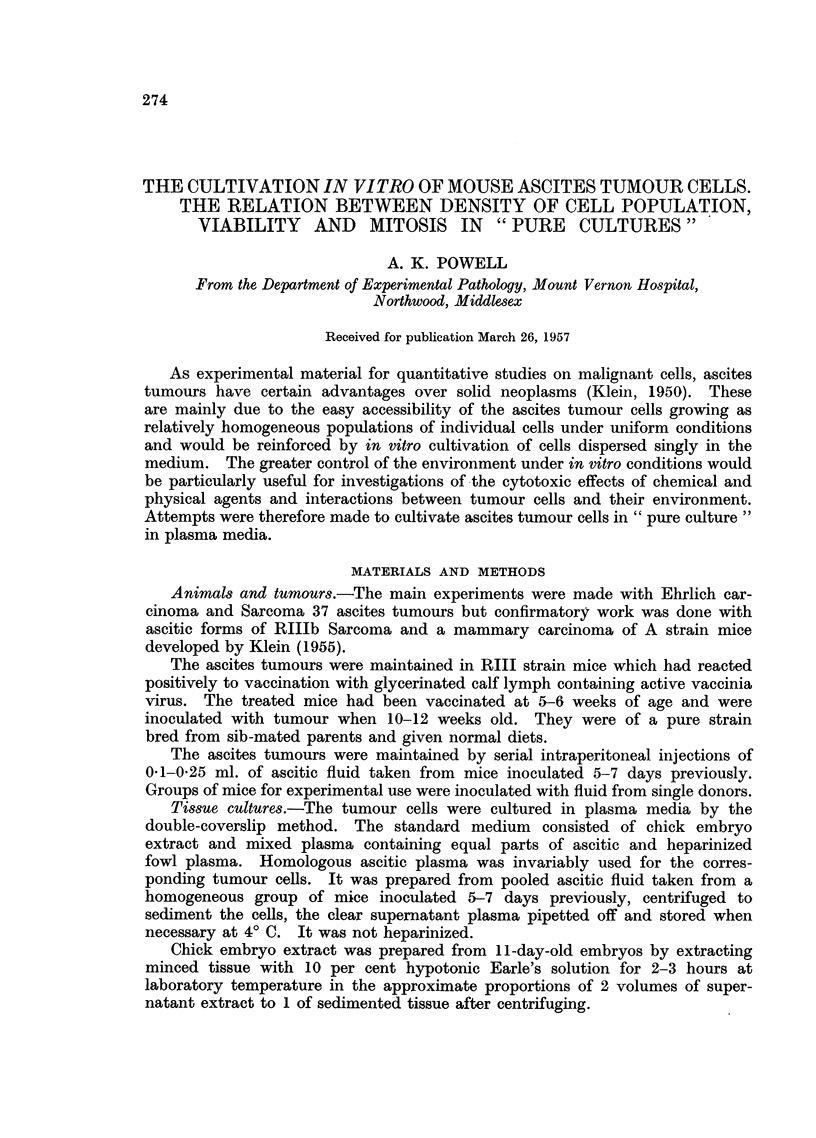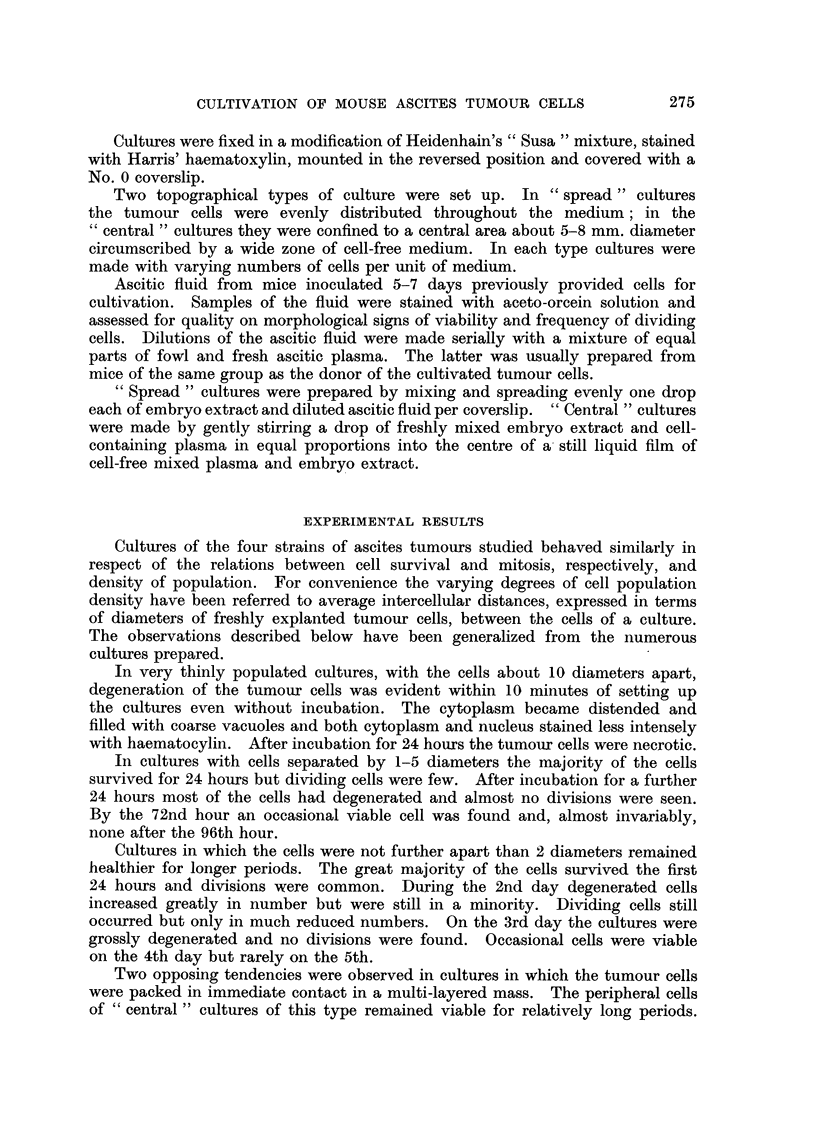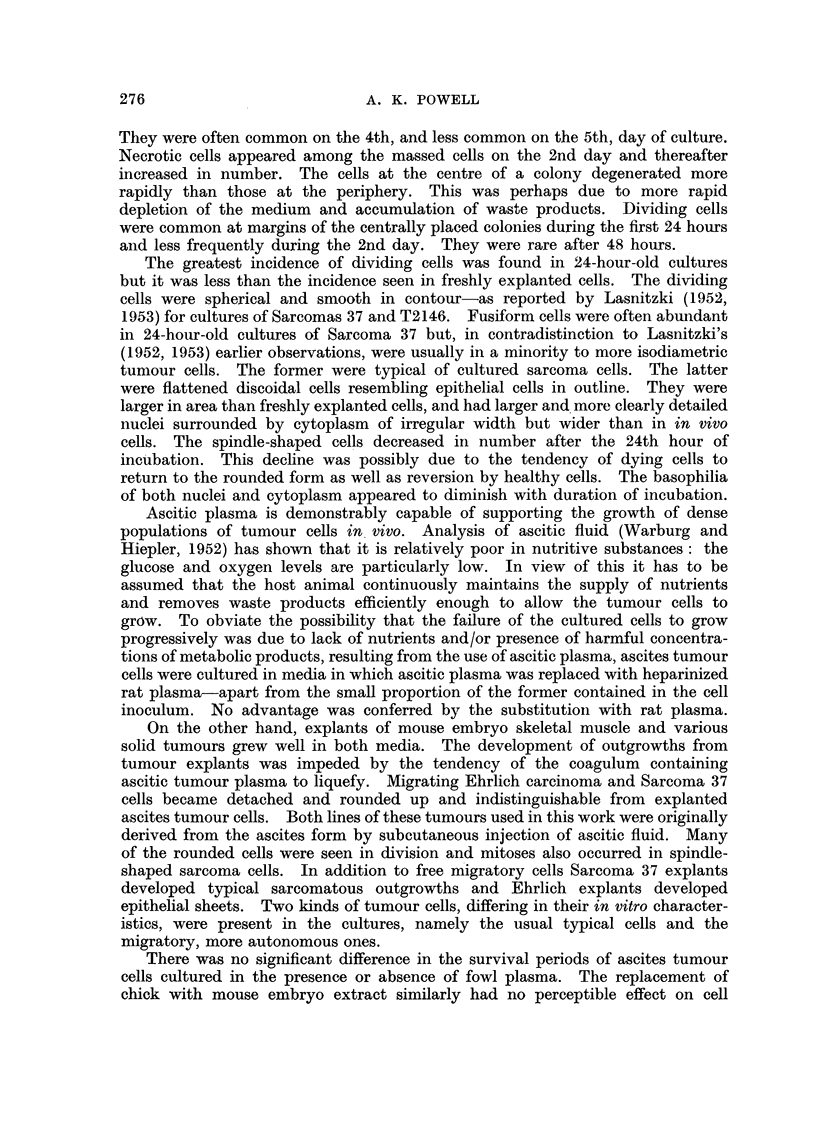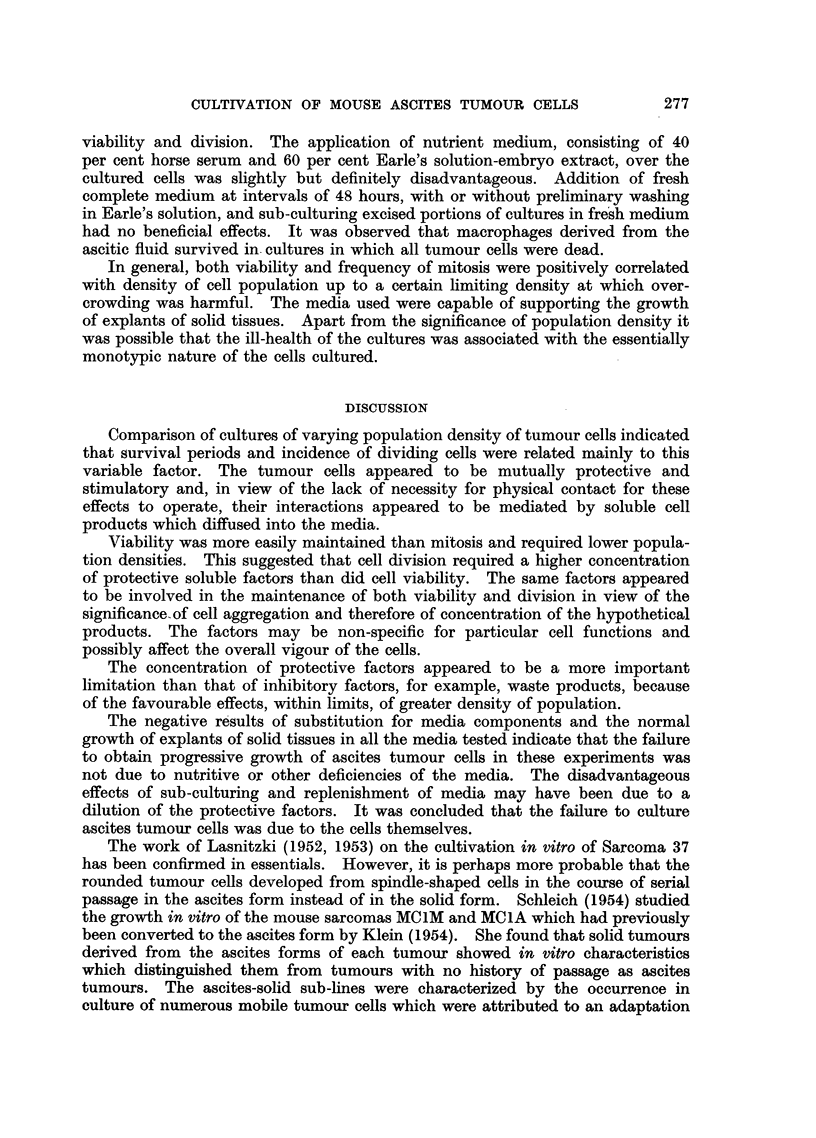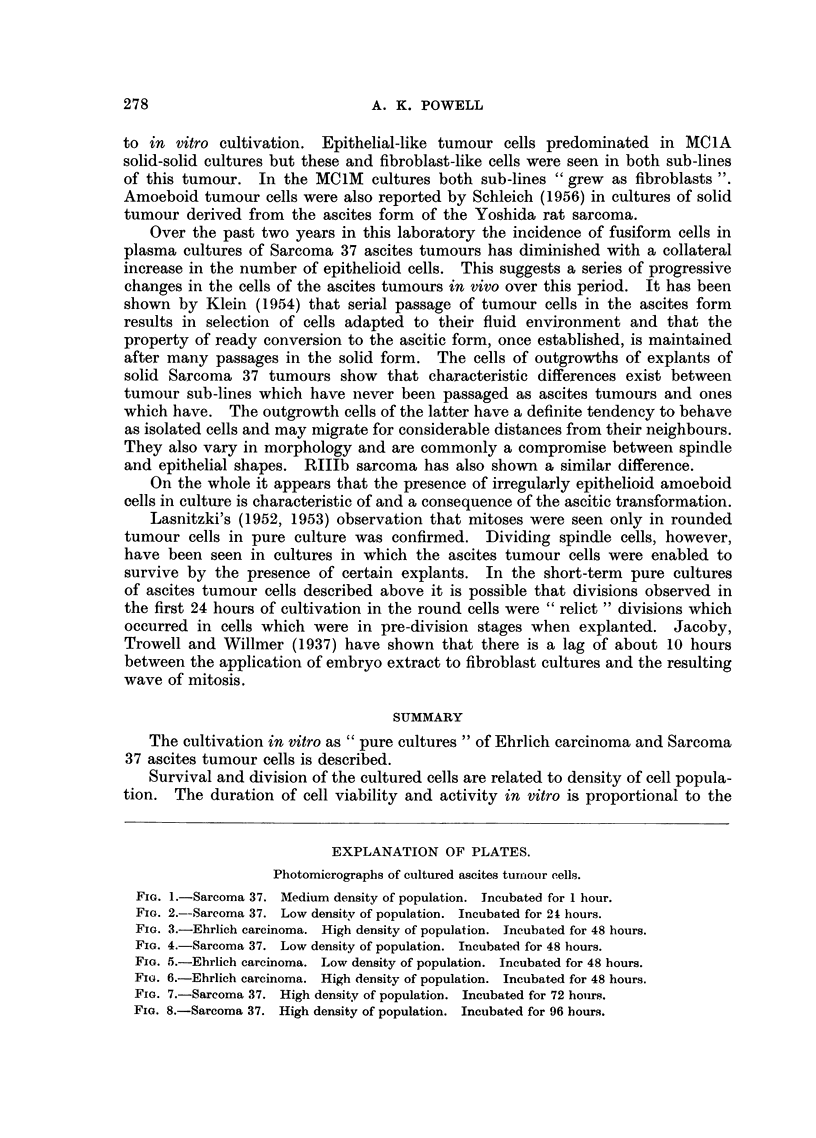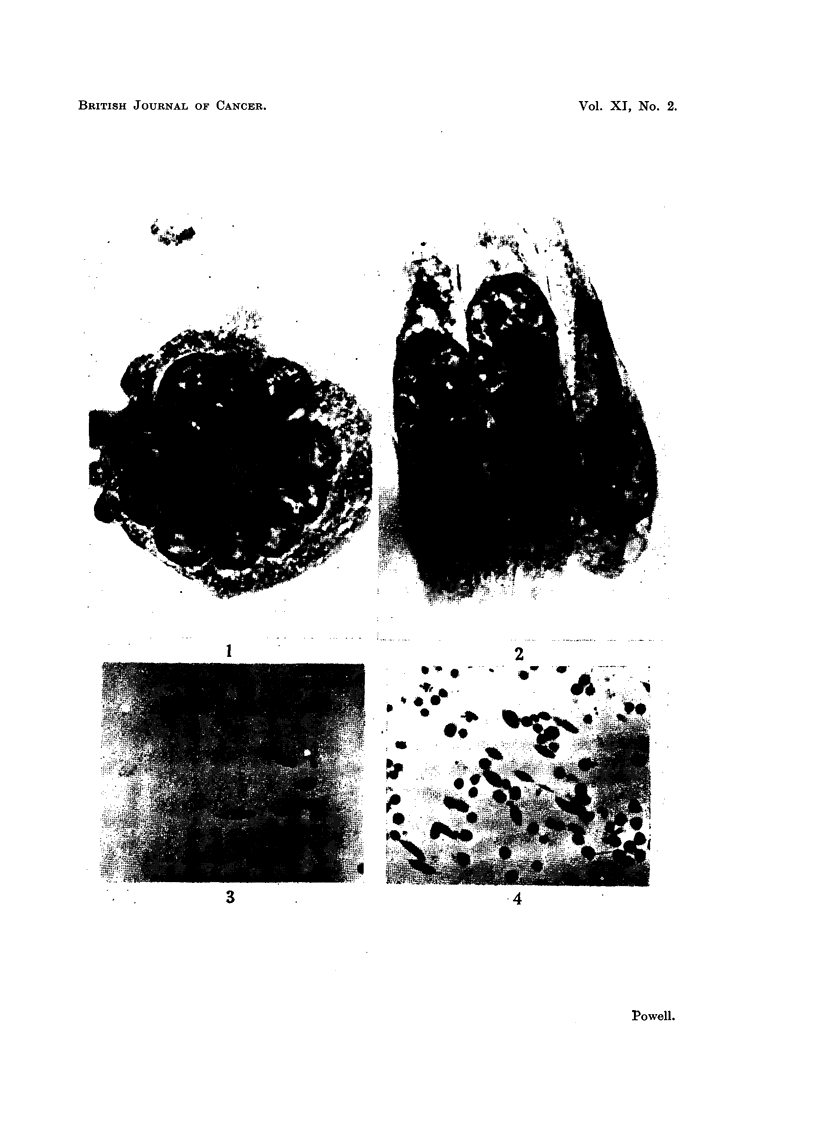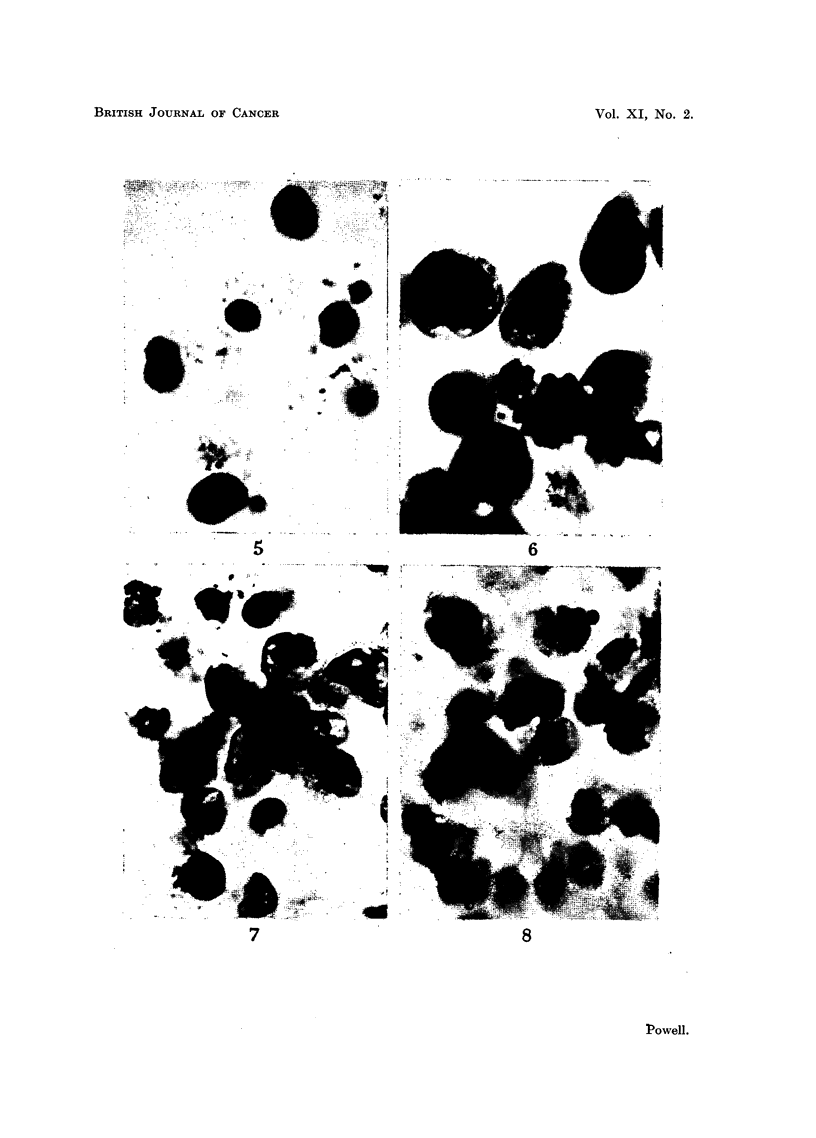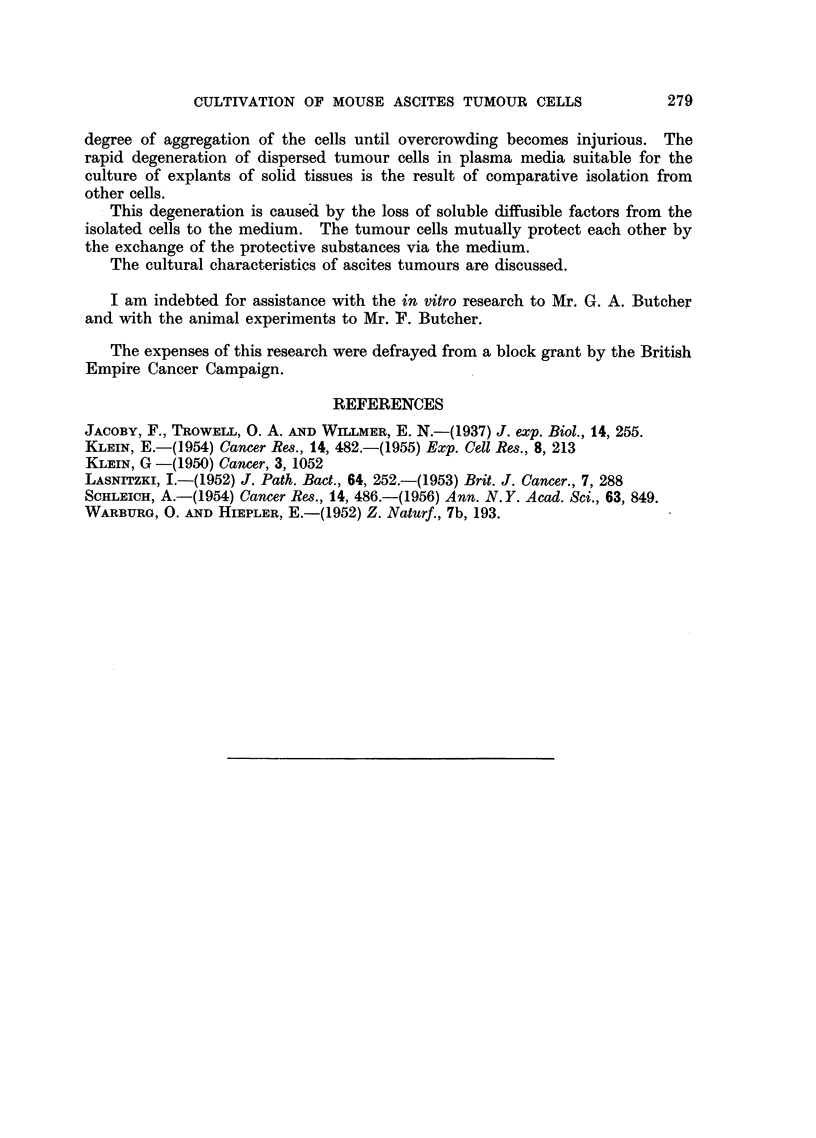# The Cultivation In Vitro of Mouse Ascites Tumour Cells. The Relation between Density of Cell Population, Viability and Mitosis in “Pure Cultures”

**DOI:** 10.1038/bjc.1957.35

**Published:** 1957-06

**Authors:** A. K. Powell

## Abstract

**Images:**


### THE CULTIVATION *IN VITRO* OF MOUSE ASCITES TUMOUR CELLS. THE RELATION BETWEEN DENSITY OF CELL POPULATION, VIABILITY AND MITOSIS IN "PURE CULTURES"

A. K. POWELL

*From the Department of Experimental Pathology, Mount Vernon Hospital, Northwood, Middlesex*

Received for publication March 26, 1957

As experimental material for quantitative studies on malignant cells, ascites tumours have certain advantages over solid neoplasms (Klein, 1950). These are mainly due to the easy accessibility of the ascites tumour cells growing as relatively homogeneous populations of individual cells under uniform conditions and would be reinforced by *in vitro* cultivation of cells dispersed singly in the medium. The greater control of the environment under *in vitro* conditions would be particularly useful for investigations of the cytotoxic effects of chemical and physical agents and interactions between tumour cells and their environment. Attempts were therefore made to cultivate ascites tumour cells in "pure culture" in plasma media.

### MATERIALS AND METHODS

*Animals and tumours.*—The main experiments were made with Ehrlich carcinoma and Sarcoma 37 ascites tumours but confirmatory work was done with ascitic forms of RIIIb Sarcoma and a mammary carcinoma of A strain mice developed by Klein (1955).

The ascites tumours were maintained in RIII strain mice which had reacted positively to vaccination with glycerinated calf lymph containing active vaccinia virus. The treated mice had been vaccinated at 5–6 weeks of age and were inoculated with tumour when 10–12 weeks old. They were of a pure strain bred from sib-mated parents and given normal diets.

The ascites tumours were maintained by serial intraperitoneal injections of 0·1–0·25 ml. of ascitic fluid taken from mice inoculated 5–7 days previously. Groups of mice for experimental use were inoculated with fluid from single donors.

*Tissue cultures.*—The tumour cells were cultured in plasma media by the double-coverslip method. The standard medium consisted of chick embryo extract and mixed plasma containing equal parts of ascitic and heparinized fowl plasma. Homologous ascitic plasma was invariably used for the corresponding tumour cells. It was prepared from pooled ascitic fluid taken from a homogeneous group of mice inoculated 5–7 days previously, centrifuged to sediment the cells, the clear supernatant plasma pipetted off and stored when necessary at 4° C. It was not heparinized.

Chick embryo extract was prepared from 11-day-old embryos by extracting minced tissue with 10 per cent hypotonic Earle's solution for 2–3 hours at laboratory temperature in the approximate proportions of 2 volumes of supernatant extract to 1 of sedimented tissue after centrifuging.

Cultures were fixed in a modification of Heidenhain's " Susa " mixture, stained with Harris' haematoxylin, mounted in the reversed position and covered with a No. 0 coverslip.

Two topographical types of culture were set up. In " spread " cultures the tumour cells were evenly distributed throughout the medium ; in the " central " cultures they were confined to a central area about 5–8 mm. diameter circumscribed by a wide zone of cell-free medium. In each type cultures were made with varying numbers of cells per unit of medium.

Ascitic fluid from mice inoculated 5–7 days previously provided cells for cultivation. Samples of the fluid were stained with aceto-orcein solution and assessed for quality on morphological signs of viability and frequency of dividing cells. Dilutions of the ascitic fluid were made serially with a mixture of equal parts of fowl and fresh ascitic plasma. The latter was usually prepared from mice of the same group as the donor of the cultivated tumour cells.

" Spread " cultures were prepared by mixing and spreading evenly one drop each of embryo extract and diluted ascitic fluid per coverslip. " Central " cultures were made by gently stirring a drop of freshly mixed embryo extract and cell-containing plasma in equal proportions into the centre of a still liquid film of cell-free mixed plasma and embryo extract.

### EXPERIMENTAL RESULTS

Cultures of the four strains of ascites tumours studied behaved similarly in respect of the relations between cell survival and mitosis, respectively, and density of population. For convenience the varying degrees of cell population density have been referred to average intercellular distances, expressed in terms of diameters of freshly explanted tumour cells, between the cells of a culture. The observations described below have been generalized from the numerous cultures prepared.

In very thinly populated cultures, with the cells about 10 diameters apart, degeneration of the tumour cells was evident within 10 minutes of setting up the cultures even without incubation. The cytoplasm became distended and filled with coarse vacuoles and both cytoplasm and nucleus stained less intensely with haematocylin. After incubation for 24 hours the tumour cells were necrotic.

In cultures with cells separated by 1–5 diameters the majority of the cells survived for 24 hours but dividing cells were few. After incubation for a further 24 hours most of the cells had degenerated and almost no divisions were seen. By the 72nd hour an occasional viable cell was found and, almost invariably, none after the 96th hour.

Cultures in which the cells were not further apart than 2 diameters remained healthier for longer periods. The great majority of the cells survived the first 24 hours and divisions were common. During the 2nd day degenerated cells increased greatly in number but were still in a minority. Dividing cells still occurred but only in much reduced numbers. On the 3rd day the cultures were grossly degenerated and no divisions were found. Occasional cells were viable on the 4th day but rarely on the 5th.

Two opposing tendencies were observed in cultures in which the tumour cells were packed in immediate contact in a multi-layered mass. The peripheral cells of " central " cultures of this type remained viable for relatively long periods.

They were often common on the 4th, and less common on the 5th, day of culture. Necrotic cells appeared among the massed cells on the 2nd day and thereafter increased in number. The cells at the centre of a colony degenerated more rapidly than those at the periphery. This was perhaps due to more rapid depletion of the medium and accumulation of waste products. Dividing cells were common at margins of the centrally placed colonies during the first 24 hours and less frequently during the 2nd day. They were rare after 48 hours.

The greatest incidence of dividing cells was found in 24-hour-old cultures but it was less than the incidence seen in freshly explanted cells. The dividing cells were spherical and smooth in contour—as reported by Lasnitzki (1952, 1953) for cultures of Sarcomas 37 and T2146. Fusiform cells were often abundant in 24-hour-old cultures of Sarcoma 37 but, in contradistinction to Lasnitzki's (1952, 1953) earlier observations, were usually in a minority to more isodiametric tumour cells. The former were typical of cultured sarcoma cells. The latter were flattened discoidal cells resembling epithelial cells in outline. They were larger in area than freshly explanted cells, and had larger and more clearly detailed nuclei surrounded by cytoplasm of irregular width but wider than in *in vivo* cells. The spindle-shaped cells decreased in number after the 24th hour of incubation. This decline was possibly due to the tendency of dying cells to return to the rounded form as well as reversion by healthy cells. The basophilia of both nuclei and cytoplasm appeared to diminish with duration of incubation.

Ascitic plasma is demonstrably capable of supporting the growth of dense populations of tumour cells *in vivo*. Analysis of ascitic fluid (Warburg and Hiepler, 1952) has shown that it is relatively poor in nutritive substances : the glucose and oxygen levels are particularly low. In view of this it has to be assumed that the host animal continuously maintains the supply of nutrients and removes waste products efficiently enough to allow the tumour cells to grow. To obviate the possibility that the failure of the cultured cells to grow progressively was due to lack of nutrients and/or presence of harmful concentrations of metabolic products, resulting from the use of ascitic plasma, ascites tumour cells were cultured in media in which ascitic plasma was replaced with heparinized rat plasma—apart from the small proportion of the former contained in the cell inoculum. No advantage was conferred by the substitution with rat plasma.

On the other hand, explants of mouse embryo skeletal muscle and various solid tumours grew well in both media. The development of outgrowths from tumour explants was impeded by the tendency of the coagulum containing ascitic tumour plasma to liquefy. Migrating Ehrlich carcinoma and Sarcoma 37 cells became detached and rounded up and indistinguishable from explanted ascites tumour cells. Both lines of these tumours used in this work were originally derived from the ascites form by subcutaneous injection of ascitic fluid. Many of the rounded cells were seen in division and mitoses also occurred in spindle-shaped sarcoma cells. In addition to free migratory cells Sarcoma 37 explants developed typical sarcomatous outgrowths and Ehrlich explants developed epithelial sheets. Two kinds of tumour cells, differing in their *in vitro* characteristics, were present in the cultures, namely the usual typical cells and the migratory, more autonomous ones.

There was no significant difference in the survival periods of ascites tumour cells cultured in the presence or absence of fowl plasma. The replacement of chick with mouse embryo extract similarly had no perceptible effect on cell viability and division. The application of nutrient medium, consisting of 40 per cent horse serum and 60 per cent Earle's solution-embryo extract, over the cultured cells was slightly but definitely disadvantageous. Addition of fresh complete medium at intervals of 48 hours, with or without preliminary washing in Earle's solution, and sub-culturing excised portions of cultures in fresh medium had no beneficial effects. It was observed that macrophages derived from the ascitic fluid survived in cultures in which all tumour cells were dead.

In general, both viability and frequency of mitosis were positively correlated with density of cell population up to a certain limiting density at which over-crowding was harmful. The media used were capable of supporting the growth of explants of solid tissues. Apart from the significance of population density it was possible that the ill-health of the cultures was associated with the essentially monotypic nature of the cells cultured.

### DISCUSSION

Comparison of cultures of varying population density of tumour cells indicated that survival periods and incidence of dividing cells were related mainly to this variable factor. The tumour cells appeared to be mutually protective and stimulatory and, in view of the lack of necessity for physical contact for these effects to operate, their interactions appeared to be mediated by soluble cell products which diffused into the media.

Viability was more easily maintained than mitosis and required lower population densities. This suggested that cell division required a higher concentration of protective soluble factors than did cell viability. The same factors appeared to be involved in the maintenance of both viability and division in view of the significance of cell aggregation and therefore of concentration of the hypothetical products. The factors may be non-specific for particular cell functions and possibly affect the overall vigour of the cells.

The concentration of protective factors appeared to be a more important limitation than that of inhibitory factors, for example, waste products, because of the favourable effects, within limits, of greater density of population.

The negative results of substitution for media components and the normal growth of explants of solid tissues in all the media tested indicate that the failure to obtain progressive growth of ascites tumour cells in these experiments was not due to nutritive or other deficiencies of the media. The disadvantageous effects of sub-culturing and replenishment of media may have been due to a dilution of the protective factors. It was concluded that the failure to culture ascites tumour cells was due to the cells themselves.

The work of Lasnitzki (1952, 1953) on the cultivation *in vitro* of Sarcoma 37 has been confirmed in essentials. However, it is perhaps more probable that the rounded tumour cells developed from spindle-shaped cells in the course of serial passage in the ascites form instead of in the solid form. Schleich (1954) studied the growth *in vitro* of the mouse sarcomas MC1M and MC1A which had previously been converted to the ascites form by Klein (1954). She found that solid tumours derived from the ascites forms of each tumour showed *in vitro* characteristics which distinguished them from tumours with no history of passage as ascites tumours. The ascites-solid sub-lines were characterized by the occurrence in culture of numerous mobile tumour cells which were attributed to an adaptation to *in vitro* cultivation. Epithelial-like tumour cells predominated in MC1A solid-solid cultures but these and fibroblast-like cells were seen in both sub-lines of this tumour. In the MC1M cultures both sub-lines " grew as fibroblasts ". Amoeboid tumour cells were also reported by Schleich (1956) in cultures of solid tumour derived from the ascites form of the Yoshida rat sarcoma.

Over the past two years in this laboratory the incidence of fusiform cells in plasma cultures of Sarcoma 37 ascites tumours has diminished with a collateral increase in the number of epithelioid cells. This suggests a series of progressive changes in the cells of the ascites tumours *in vivo* over this period. It has been shown by Klein (1954) that serial passage of tumour cells in the ascites form results in selection of cells adapted to their fluid environment and that the property of ready conversion to the ascitic form, once established, is maintained after many passages in the solid form. The cells of outgrowths of explants of solid Sarcoma 37 tumours show that characteristic differences exist between tumour sub-lines which have never been passaged as ascites tumours and ones which have. The outgrowth cells of the latter have a definite tendency to behave as isolated cells and may migrate for considerable distances from their neighbours. They also vary in morphology and are commonly a compromise between spindle and epithelial shapes. RIIIb sarcoma has also shown a similar difference.

On the whole it appears that the presence of irregularly epithelioid amoeboid cells in culture is characteristic of and a consequence of the ascitic transformation.

Lasnitzki's (1952, 1953) observation that mitoses were seen only in rounded tumour cells in pure culture was confirmed. Dividing spindle cells, however, have been seen in cultures in which the ascites tumour cells were enabled to survive by the presence of certain explants. In the short-term pure cultures of ascites tumour cells described above it is possible that divisions observed in the first 24 hours of cultivation in the round cells were " relict " divisions which occurred in cells which were in pre-division stages when explanted. Jacoby, Trowell and Willmer (1937) have shown that there is a lag of about 10 hours between the application of embryo extract to fibroblast cultures and the resulting wave of mitosis.

### SUMMARY

The cultivation *in vitro* as " pure cultures " of Ehrlich carcinoma and Sarcoma 37 ascites tumour cells is described.

Survival and division of the cultured cells are related to density of cell population. The duration of cell viability and activity *in vitro* is proportional to the

---

### EXPLANATION OF PLATES.

Photomicrographs of cultured ascites tumour cells.

FIG. 1.—Sarcoma 37. Medium density of population. Incubated for 1 hour.
FIG. 2.—Sarcoma 37. Low density of population. Incubated for 24 hours.
FIG. 3.—Ehrlich carcinoma. High density of population. Incubated for 48 hours.
FIG. 4.—Sarcoma 37. Low density of population. Incubated for 48 hours.
FIG. 5.—Ehrlich carcinoma. Low density of population. Incubated for 48 hours.
FIG. 6.—Ehrlich carcinoma. High density of population. Incubated for 48 hours.
FIG. 7.—Sarcoma 37. High density of population. Incubated for 72 hours.
FIG. 8.—Sarcoma 37. High density of population. Incubated for 96 hours.

1

2

3

4

5

6

7

8

degree of aggregation of the cells until overcrowding becomes injurious. The rapid degeneration of dispersed tumour cells in plasma media suitable for the culture of explants of solid tissues is the result of comparative isolation from other cells.

This degeneration is caused by the loss of soluble diffusible factors from the isolated cells to the medium. The tumour cells mutually protect each other by the exchange of the protective substances via the medium.

The cultural characteristics of ascites tumours are discussed.

I am indebted for assistance with the *in vitro* research to Mr. G. A. Butcher and with the animal experiments to Mr. F. Butcher.

The expenses of this research were defrayed from a block grant by the British Empire Cancer Campaign.

### REFERENCES

JACOBY, F., TROWELL, O. A. AND WILLMER, E. N.—(1937) *J. exp. Biol.*, **14**, 255.
KLEIN, E.—(1954) *Cancer Res.*, **14**, 482.—(1955) *Exp. Cell Res.*, **8**, 213
KLEIN, G —(1950) *Cancer*, **3**, 1052
LASNITZKI, I.—(1952) *J. Path. Bact.*, **64**, 252.—(1953) *Brit. J. Cancer.*, **7**, 288
SCHLEICH, A.—(1954) *Cancer Res.*, **14**, 486.—(1956) *Ann. N.Y. Acad. Sci.*, **63**, 849.
WARBURG, O. AND HIEPLER, E.—(1952) *Z. Naturf.*, 7b, 193.